# SIRT7‐Mediated MVP Desuccinylation Facilitates Tongue Squamous Cell Carcinoma Progression by Activating JAK2/STAT3 Pathway

**DOI:** 10.1002/cbin.70048

**Published:** 2025-06-30

**Authors:** Zhuo Zhang, Tingting Guo, Xiangyu Zhao

**Affiliations:** ^1^ Department of Stomatology The First Affiliated Hospital of JinZhou Medical University JinZhou China; ^2^ Beihua University Jilin China; ^3^ The Department of Comprehensive Emergency Stomatological Hospital of Shenyang Shenyang China

**Keywords:** JAK2/STAT3 pathway, MVP, progression, SIRT7, tongue squamous cell carcinoma

## Abstract

Major vault protein (MVP) plays a contributing role in multifarious cancers, and then its role in Tongue squamous cell carcinoma (TSCC) is uncomprehending. This study aimed to investigate the regulatory effect of MVP on malignant behavior of TSCC cells and its mechanism. We first pointed out the abnormal upregulation of MVP in tumor tissues by immunohistochemistry, western blot, and reverse transcription‐quantitative polymerase chain reaction assays. Depletion of MVP hindered TSCC cell viability, migration, and invasion and accelerated apoptosis. Mechanistically, depletion of MVP inactivated Janus Kinase 2 (JAK2)/Signal Transducer and Activator of Transcription 3 (STAT3) pathway. Coumermycin A1 (CA1), a JAK2 agonist, was used to trigger JAK2/STAT3 signaling. Functional experiments demonstrated that CA1 significantly counteracted the inhibitory effects of MVP silencing on cell proliferation, invasion, and migration, as well as the stimulatory effects of MVP silencing on cell apoptosis. Moreover, we discovered that MVP undergoes succinylation and identified Sirtuin 7 (SIRT7) as the desuccinylase for MVP. Addition of SIRT7 promoted the protein stability of MVP in TSCC cells. Further, addition of MVP expedited the viability, migration, and invasion and suppressed apoptosis of TSCC cells, which was partly neutralized following depleted SIRT7. Our findings revealed that MVP desuccinylated by SIRT7 accelerated TSCC progression via regulating JAK2/STAT3 signaling.

**Abbreviations**
CA1: Coumermycin A1
CCK‐8: cell counting kit‐8
ccRCC: clear cell renal cell carcinoma
CHX: cycloheximide
Co‐IP: co‐immunoprecipitation
HNSC: squamous cell carcinoma of the head and neck
IHC: immunohistochemistry
JAK2: Janus Kinase 2
MVP: major vault protein
RT‐qPCR: reverse transcription‐quantitative polymerase chain reaction
SIRT7: sirtuin 7
STAT3: signal transducer and activator of transcription 3
TSCC: tongue squamous cell carcinoma

## 1 Introduction

Squamous cell carcinoma of the head and neck (HNSC) is one of the most common cancers in the world, with over 600,000 new confirmed cases each year (Lenze et al. 2020). In many developed countries, the incidence rate of most head and neck cancers has been declining with the reduction of tobacco consumption (Johnson et al. 2020). However, the incidence rate of oral tongue squamous cell carcinoma (TSCC), a subtype of head and neck cancer, is on the rise, especially the incidence rate of young men and women (< 45 years old at the time of diagnosis) has the largest increase (Bray et al. 2018). TSCC occurs in the oral mucosa and has rapid local infiltration and migration, and the occurrence of this tumor is related to smoking, alcohol abuse, and human papillomavirus infection (Siegel et al. 2020). Regardless of the presence or absence of neck lymph node dissection, surgical resection of primary TSCC is still considered the gold standard for its treatment, while other treatment methods include systemic therapy (chemotherapy or targeted therapy) and/or radiotherapy (Colevas et al. 2018; Hussein et al. 2018). Unfortunately, TSCC patients are prone to local recurrence and metastasis after primary treatment, making it difficult to treat, with a 5‐year survival rate of only 50% (Hussein et al. 2018; Colella et al. 2021). Therefore, studying the mechanism of TSCC progression will help to develop new and effective targets for TSCC treatment.

The human gene encoding major vault protein (MVP) is located on chromosome 16 (16p11.2), with a distance of about 27 cm from the multiple drug resistance protein‐1 (MRP1) gene (Slovak et al. 1995). It has been confirmed that MVP expression is related to occurrence of drug resistance in cancer cells (Scheffer et al. 1995). Moreover, MVP has also been revealed to promote hepatocellular carcinoma progression by targeting IRF2 and reducing p53 activity (Yu et al. 2020). Also, MVP could facilitate glioblastoma cell survival and migration by activating the EGFR/PI3K signaling (Lötsch et al. 2013). Silva et al. found that MVP expression was closely related to local treatment failure and cancer‐specific survival in oropharyngeal squamous cell carcinoma, supporting that MVP may be a prognostic marker associated with radiation resistance (Silva et al. 2007). Overexpression of MVP is closely related to poor disease‐free survival and etiology‐specific survival in patients with oral squamous cell carcinoma at stage III‐IV (Henríquez‐Hernández et al. 2012). However, the function and related molecular mechanisms of MVP in TSCC have been less explored.

This study verified the function of MVP in TSCC and laid a theoretical foundation for finding new molecular targets for TSCC treatment.

## 2 Methods and Materials

### 2.1 Bioinformatics Analysis

HitPredict (https://www.hitpredict.org/index.html) and BIOGRID databases (https://thebiogrid.org/) are used to predict MVP‐binding proteins. GPSuc database and SuccinSite database were employed to screen succinylation sites of MVP.

### 2.2 Patients and Clinical Specimens

The tumor and adjacent normal tissues used in this study were obtained from 30 patients with TSCC at the Stomatological Hospital of Shenyang. Tissue samples taken from patients are immediately frozen in liquid nitrogen. Informed consent forms were signed and collected by all patients. This study was approved by the Stomatological Hospital of Shenyang (China).

### 2.3 Cell Culture and Transfection

Normal human oral keratinocyte cell line (NHOK) and TSCC cell lines (CAL‐27, SCC9, and SCC25) were obtained from ATCC (Virginia, USA). All the cell lines were cultured in DMEM with 10% FBS and 1% penicillin‐streptomycin antibiotics.

Short hairpin RNA (shRNA) targeting MVP or SIRT7 (sh‐MVP#1/#2/#3 or sh‐SIRT7) and shNC as control, overexpression plasmids containing MVP (pcDNA3.1‐MVP) and empty pcDNA3.1 vector as control, were synthesized and provided by Shanghai Genepharm. Cell transfection was conducted using Lipofectamine 2000 Reagent. Cells were subjected to subsequent experiments 48 h after the transfection.

### 2.4 Reverse Transcription‐Quantitative Polymerase Chain Reaction (RT‐qPCR)

Total RNA from CAL‐27 and SCC9 cells was separated employing the TRIzol‐based method (Invitrogen) and reverse‐transcribed into cDNA using the RevertAid RT Kit (Thermo Fisher Scientific). The qPCR was performed on SYBR Green Master Mix. Gene expression quantification was assessed using the 2^−ΔΔCt^ method.

### 2.5 Western Blot Assay

CAL‐27 and SCC9 cells were purified using RIPA lysis buffer and quantified using a BCA Protein Assay Kit (Beyotime). Total protein (20 μg) was subjected to 10% SDS‐PAGE, transferred to PVDF membranes, and hybridized with corresponding primary antibodies at 4°C overnight, followed by secondary antibodies. After that, protein signal was visualized through a chemiluminescence ECL kit and GAPDH as load control.

### 2.6 Co‐Immunoprecipitation (Co‐IP)

CAL‐27 and SCC9 cells were lysed in IP lysis buffer (Beyotime, China) containing protease and phosphatase inhibitors (Roche, Switzerland) on ice for 30 min. The lysates were centrifuged at 12,000 × *g* for 15 min at 4°C to remove debris. The supernatants were collected and precleared with 20 μL of protein A/G magnetic beads (MedChemExpress, USA) for 1 h at 4°C with rotation. After preclearing, the supernatants (500 μg of total protein) were incubated overnight at 4°C with 2 μg of the indicated primary antibodies (anti‐SIRT7, anti‐MVP, or control IgG). The next day, 30 μL of protein A/G magnetic beads were added and incubated for another 2 h at 4°C with gentle rotation to pull down the immune complexes. The beads were then washed five times with cold lysis buffer. The bound proteins were eluted by boiling in SDS loading buffer for 5 min and analyzed by western blotting analysis using the indicated antibodies. Input (10% of total lysate) was included as a positive control.

### 2.7 Cell Counting Kit‐8 (CCK‐8)

Cell proliferation was evaluated using the CCK‐8 assay (Dojindo, Japan). CAL‐27 and SCC9 cells were seeded into 96‐well plates at a density of 2 × 10³ cells/well in 100 μL of complete medium. At 0, 24, 48, and 72 h postseeding, 10 μL of CCK‐8 solution was added to each well and incubated for 2 h at 37°C. The absorbance at 450 nm was measured using a microplate reader (Bio‐Rad, USA). Each condition was tested in triplicate.

### 2.8 Wound Healing Assay

CAL‐27 and SCC9 cells were seeded in 6‐well plates at a density of 5 × 10⁵ cells/well and cultured until reaching ~90% confluence. A sterile 200‐μL pipette tip was used to create a linear scratch across the cell monolayer. Cells were washed twice with PBS to remove detached cells and incubated in serum‐free DMEM for 24 h. Images of the wound area were captured at 0 and 24 h using an inverted microscope (Olympus, Japan). The wound closure rate was calculated using the following formula: Wound healing (%) = [(*W₀* − *W*_t_)/*W*₀] × 100%. where *W₀* represents the initial wound width at 0 h, and *W*_t_ represents the remaining wound width at 24 h. The wound widths were measured using ImageJ software, and the relative closure percentage was used to assess cell migration efficiency.

### 2.9 Transwell Assay

The invasive ability of CAL‐27 and SCC9 cells was evaluated using 24‐well Transwell chambers with 8.0 μm pore‐size polycarbonate membrane inserts (Corning, USA) pre‐coated with Matrigel (BD Biosciences, USA). Briefly, the upper surface of the inserts was coated with 50 μL of Matrigel (diluted 1:8 in serum‐free medium) and incubated at 37°C for 1 h to allow gelation. Cells were harvested, resuspended in serum‐free DMEM, and seeded into the upper chambers at a density of 1 × 10⁵ cells in 200 μL per well. The lower chambers were filled with 500 μL of DMEM containing 10% fetal bovine serum (FBS) as a chemoattractant. After 24 h of incubation at 37°C, the non‐invaded cells on the upper surface were gently removed with a cotton swab. The invaded cells on the lower surface were fixed with 4% paraformaldehyde for 20 min and stained with 0.1% crystal violet (Sangon Biotech, China) for 15 min. The number of invaded cells was counted under an inverted microscope (Olympus) in five randomly selected fields per insert.

## 3 Cell Apoptosis Analysis

Apoptosis was assessed using Annexin V‐APC/PI staining (BD Biosciences, USA) according to the manufacturer's protocol. CAL‐27 and SCC9 cells were harvested, washed with PBS, and resuspended in 1× binding buffer at a concentration of 1 × 10⁷ cells/mL. For each sample, 1 × 10⁶ cells were incubated with 5 μL Annexin V‐APC and 5 μL PI staining solution in the dark for 15 min at room temperature. The apoptotic cells were quantified by flow cytometry using a BD FACSCalibur system, and the data were analyzed with FlowJo software.

### 3.1 Cycloheximide (CHX) Assay

To assess the effect of SIRT7 on MVP protein stability, a CHX chase assay was performed. CAL‐27 and SCC9 cells were seeded in 6‐well plates at a density of 4 × 10⁵ cells/well and cultured overnight. Cells were transfected with either oe‐NC or oe‐SIRT7 expression vectors using Lipofectamine 3000 (Invitrogen, USA) according to the manufacturer's protocol. After 24 h of transfection, cells were treated with 50 μg/mL CHX (CHX; Sigma‐Aldrich, USA) to inhibit protein synthesis. Cells were harvested at 0, 6, 12, and 24 h following CHX treatment. At each time point, total protein was extracted using RIPA lysis buffer (Beyotime, China) supplemented with protease inhibitors (Roche, Switzerland). Protein concentrations were quantified using a BCA protein assay kit (Thermo Fisher Scientific, USA). Equal amounts of protein were subjected to SDS‐PAGE followed by western blot analysis to detect MVP protein levels.

### 3.2 Injection of Tumor Cells Into Nude Mice

Male BALB/C nude mice were obtained from Beijing Vital River Laboratory Animal Technology Co. Ltd. The mice were housed in a pathogen‐free laminar flow cabinet, provided with unrestricted access to food and high‐pressure water, and kept on a 12‐h dark/light cycle. CAL‐27 cells were stably transduced with a lentiviral vector carrying the firefly luciferase gene (Luc) (GeneChem, Shanghai, China), and positive clones were selected using puromycin (2 μg/mL) before injection into nude mice. The experiment was divided into two groups (sh‐NC and sh‐MVP). The left forelimb of mice in each group was injected with 100 μL of CAL‐27 cells (5.0 × 10^7^ cells/mL) according to their respective groups. The length and width of each neoplasm were measured on Day 9. After 30 days, BALB/C nude mice in each group were euthanized, and the tumor weight and volume were weighed. The study was approved by the Ethics Committee of Stomatological Hospital of Shenyang.

### 3.3 Tumor Tissue Staining

The tumors in each group were fixed with 4% neutral paraformaldehyde, dehydrated with ethanol, permeated with xylene, and embedded in paraffin. Then, 5 μm tumor sections were prepared. H&E staining was used to observe the pathological changes of tumors.

For Immunohistochemistry (IHC) analysis, TSCC tissue fixation, paraffin embedding. Tissues were incubated with anti‐Ki‐67 overnight, and then incubated with a secondary antibody. The tissues were stained with 3, 3‐diaminobenzidine solution and hematoxylin. The tissues were observed under a microscope.

### 3.4 Mass Spectrometry Analyses

Total cell lysates of CAL‐27 cells transfected with MVP‐Flag were incubated with anti‐flag M2 magnetic beads for more than 4 h at 4°C. The protein‐bead complexes were washed three times with ice‐cold NP40 buffer. Then, the proteins were eluted from beads by incubating 3×Flag peptides solution at 4°C for 2 h. Finally, total eluted proteins were assessed by mass spectrometry in PTM Biolabs (Hangzhou, China).

### 3.5 Statistical Analysis

Independent experiments were repeated three times. Continuous variables were expressed as means ± standard deviation. Graphing and analysis of data were done by using GraphPad Prism 8 with one‐way ANOVA or two‐tailed Student's t‐test. Significance levels were set at *p* < 0.05.

## 4 Results

### 4.1 MVP Expression Was Elevated in TSCC Tissues and Cells

**Figure 1:**
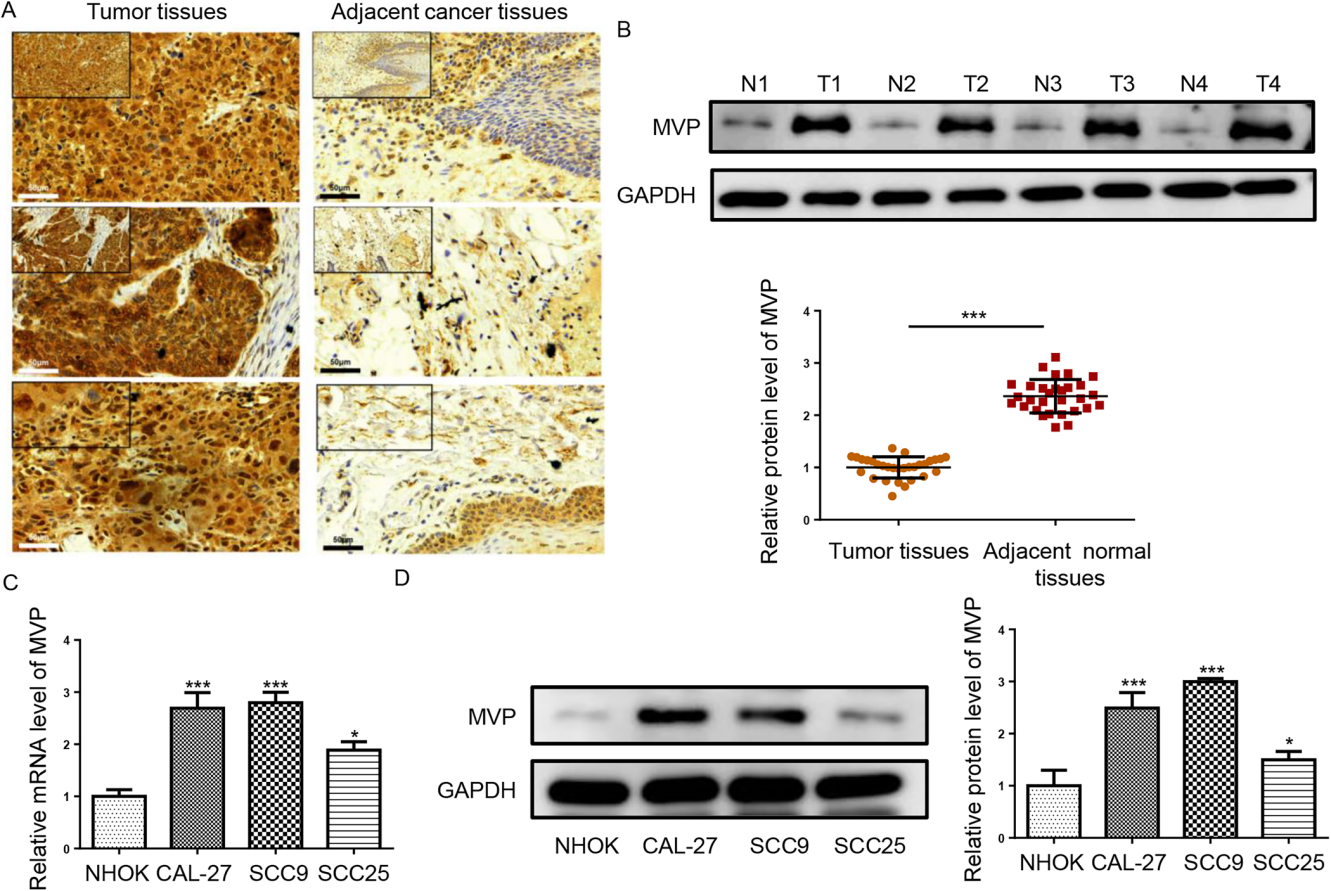
MVP expression was elevated in TSCC tissues and cells. (A) IHC (scale bars = 50 μm) was used to detect MVP expression in TSCC samples (*n* = 30) and adjacent normal tissues (*n* = 30). (B and C) RT‐qPCR and Western blot assays were performed to measure MVP mRNA and protein levels in TSCC tissues. (D and E) RT‐qPCR and Western blot assays were performed to measure MVP mRNA and protein levels in TSCC cells (CAL‐27, SCC9, and SCC25) and NHOK cells. *p < 0.05; **p < 0.01; ***p < 0.001.

To explore the relationship between MVP expression and TSCC, we employed IHC to perform MVP staining on TSCC samples and adjacent normal tissues. Figure 1A demonstrated significantly elevated levels of MVP in TSCC tissues. Western blot and RT‐qPCR results also showed that MVP level was highly expressed in TSCC tissues (Figure 1B,C). Moreover, MVP expression was enhanced in TSCC cells (CAL‐27, SCC9, and SCC25) compared to NHOK cells (Figure 1D,E). These data suggest that MVP might play an oncogenic role in TSCC.

### 4.2 Inhibition of MVP Expression Impeded TSCC Cell Malignant Behaviors

**Figure 2:**
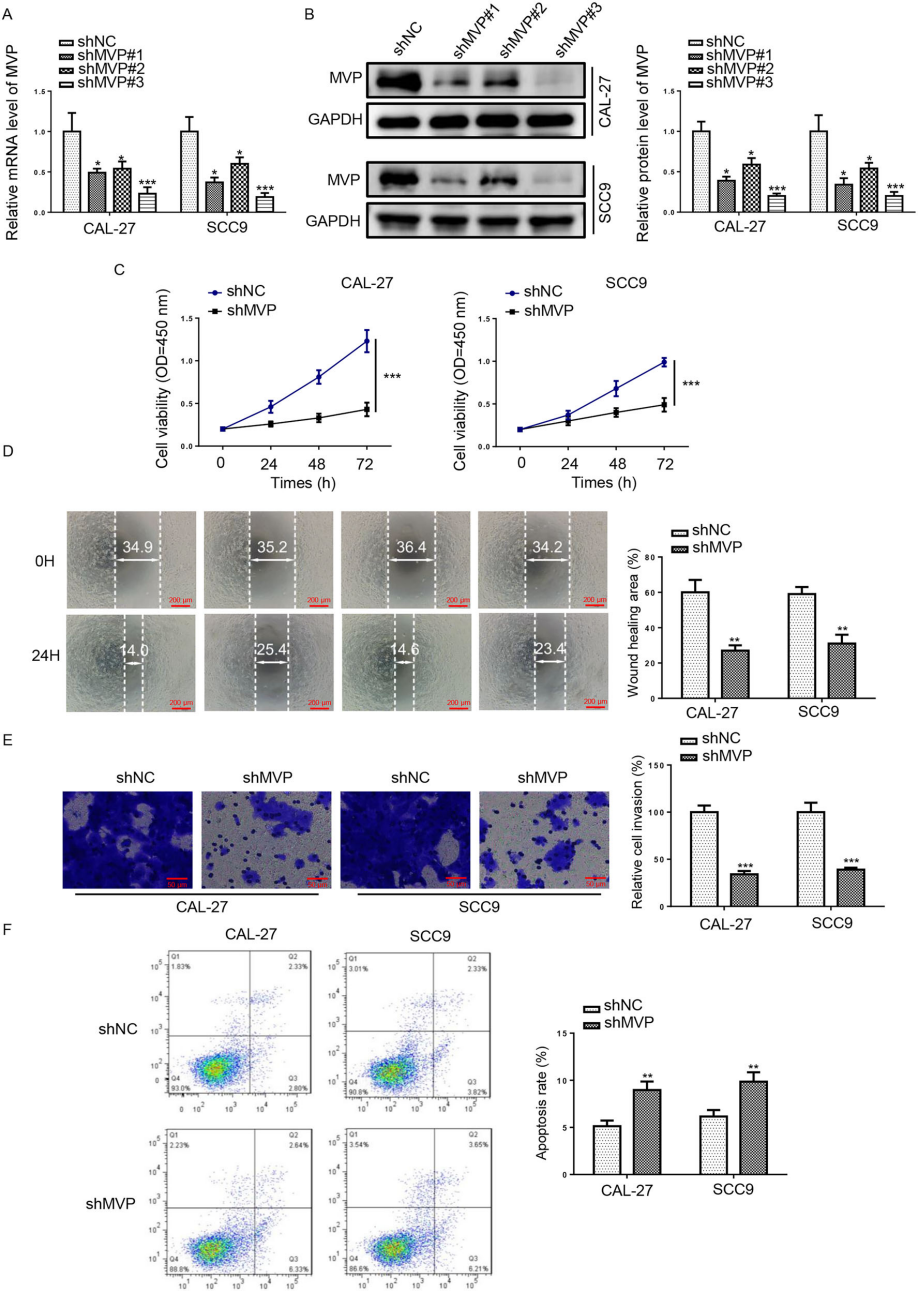
Inhibition of MVP expression impeded TSCC cell malignant behaviors. (A and B) RT‐qPCR and Western blot assays were used to detect MVP mRNA and protein levels in TSCC cells transfected with sh‐NC, sh‐MVP#1, sh‐MVP#2, or sh‐MVP#3. (C) CCK‐8 assay was performed to measure cell viability of TSCC cells transfected with shNC or sh‐MVP. (D and E) Wound healing (scale bars = 200 μm) and transwell assays (scale bars = 50 μm) were used to measure cell migration and invasion in TSCC cells. (F) Cell apoptosis and cell cycle were assessed by flow cytometry assay. *p < 0.05; **p < 0.01; ***p < 0.001.

To delve deeper into the function of MVP in TSCC advancement, we constructed shRNA of MVP (sh‐MVP#1/#2/#3). As depicted in Figure 2A,B, the mRNA and protein levels of MVP were suppressed by MVP depletion (sh‐MVP#3). sh‐MVP#3 had the highest knockout efficiency, so sh‐MVP#3 (sh‐MVP) was used in subsequent experiments. Then, CCK‐8 results indicated a notable dampening of proliferation in CAL‐27 and SCC9 cells following MVP depletion (Figure 2C). Moreover, MVP deficiency impeded TSCC cell migration and invasive abilities (Figure 2D,E). Besides, flow cytometry assay results exhibited that MVP silencing increased apoptotic cell numbers and led to G1 arrest (Figure 2F,G). Collectively, these discoveries manifested that MVP deficiency curbed the malignant behavior of TSCC cells.

### 4.3 MVP Promoted TSCC Growth In Vivo

**Figure 3:**
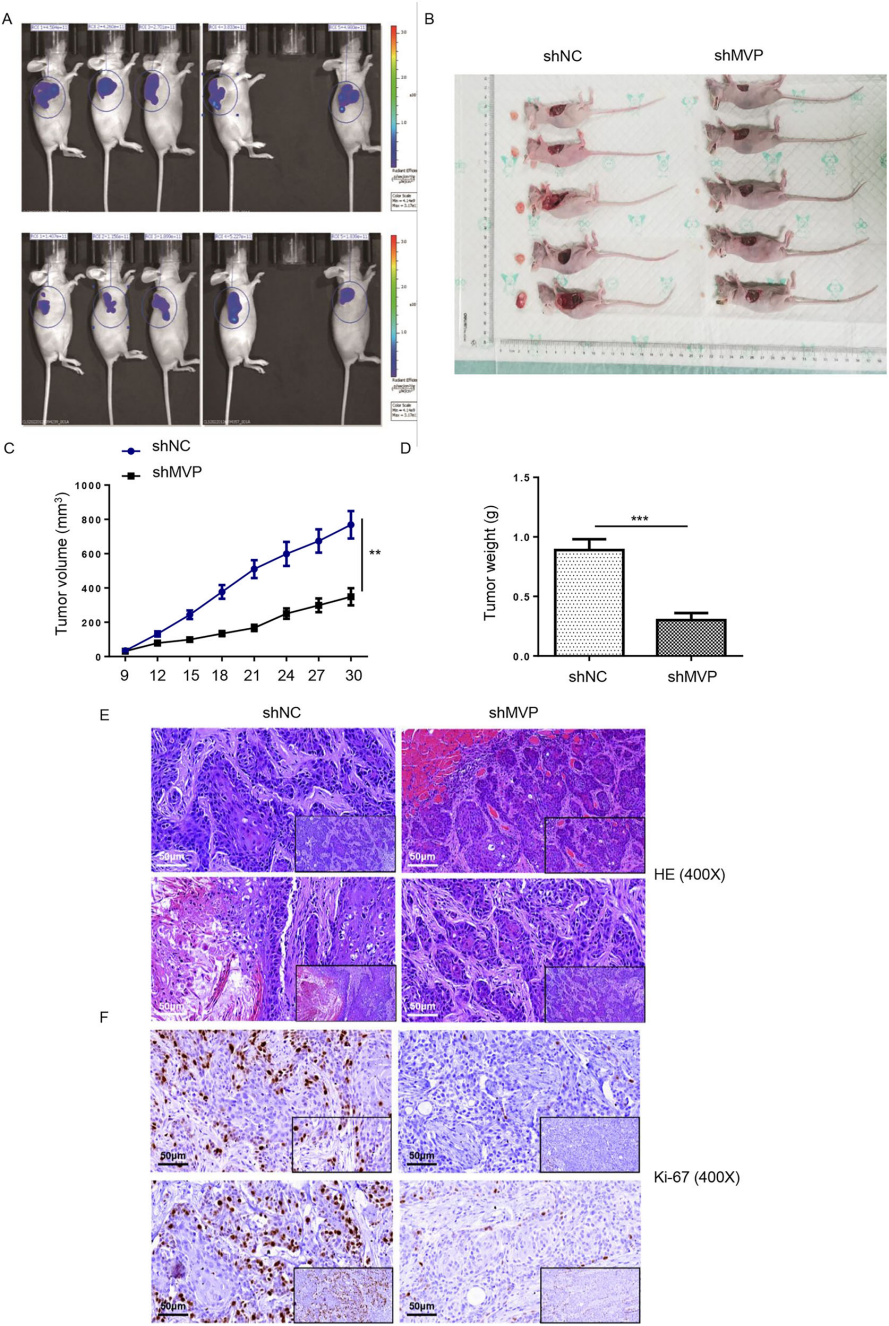
MVP promoted TSCC growth in vivo. (A) In vivo imaging was performed to evaluate the tumor burden in mice of sh‐MVP and sh‐NC groups. (B) Tumors were removed for collecting photos and weighing (C). (D) The volume of tumors formed in mice was measured and calculated at indicated time intervals. (E and F) HE and ki‐67 staining of tumors (scale bars = 50 μm). *p < 0.05; **p < 0.01; ***p < 0.001.

Mouse models were constructed by injecting CAL‐27 cells (with or without MVP silencing). As illuminated in Figure 3A, the luminescent intensity was significantly weaker in the sh‐MVP group. Depletion of MVP decreased tumor growth, as evidenced by tumor size, volume, weight, HE, and Ki67 staining (Figure 3B–F). Collectively, knockdown of MVP suppressed tumor growth of TSCC in vivo.

### 4.4 Depletion of MVP Suppressed the Activation of JAK2/STAT3 Pathway in TSCC

**Figure 4:**
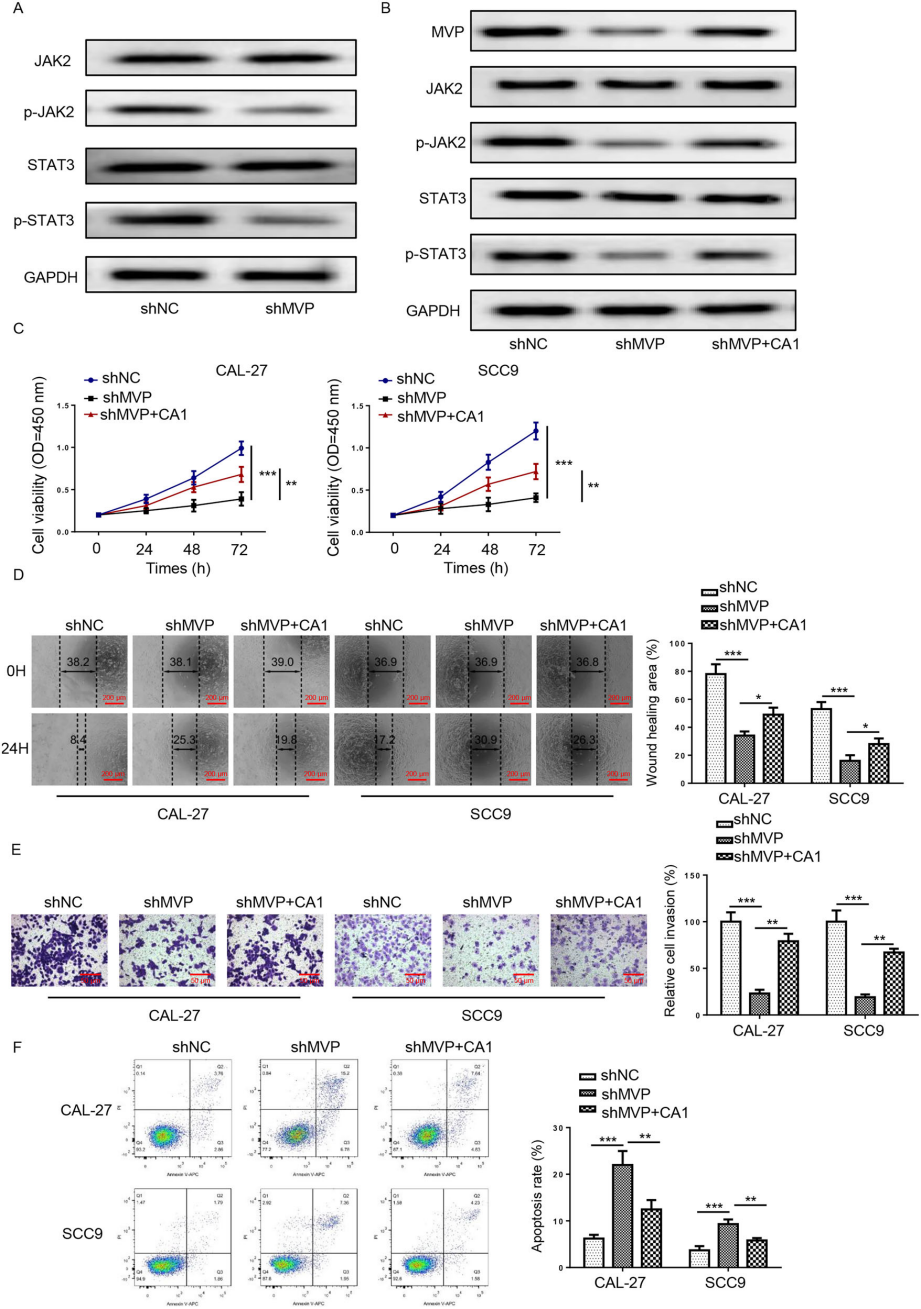
Depletion of MVP suppressed the activation of JAK2/STAT3 pathway in TSCC. (A) p‐JAK2, p‐STAT3, JAK2, and STAT3 protein levels were measured by western blot assay in TSCC cells transfected with shNC or sh‐MVP. The experiment was divided into the following groups: sh‐NC, sh‐MVP, sh‐MVP + CA1. (B) p‐JAK2, p‐STAT3, JAK2, and STAT3 protein levels were measured by western blot assay in TSCC cells. (C) Cell proliferation, (D) migration (scale bars=200μm), (E) invasion (scale bars = 50 μm), and (F) apoptosis were measured by CCK‐8, wound healing, transwell, and flow cytometry assays in TSCC cells. *p < 0.05; **p < 0.01; ***p < 0.001.

Next, we investigated whether JAK/STAT signaling was implicated in MVP‐induced TSCC progression. As demonstrated in Figure 4A, MVP silencing distinctly declined p‐JAK2 and p‐STAT3 protein levels, suggesting that MVP/JAK2/STAT3 axis plays a vital role in the TSCC development.

To expound whether MVP aggravates TSCC cell malignant behaviors by activating JAK2/STAT3 signaling, CA1, a JAK2 agonist, was applied to activate the JAK2/STAT3 pathway with or without MVP silencing. CA1 eliminated sh‐MVP‐mediated downregulation of p‐JAK2 and p‐STAT3 protein levels (Figure 4B). Moreover, CA1 reversed the effects of sh‐MVP on cell proliferation, migration, invasion, and apoptosis (Figure 4C–F) in TSCC cells. The above results indicated that MVP promotes TSCC cell malignant behaviors via regulating JAK2/STAT3 axis.

### 4.5 Depletion of SIRT7 Enhanced MVP‐SUCC Level in Tscc Cells

**Figure 5:**
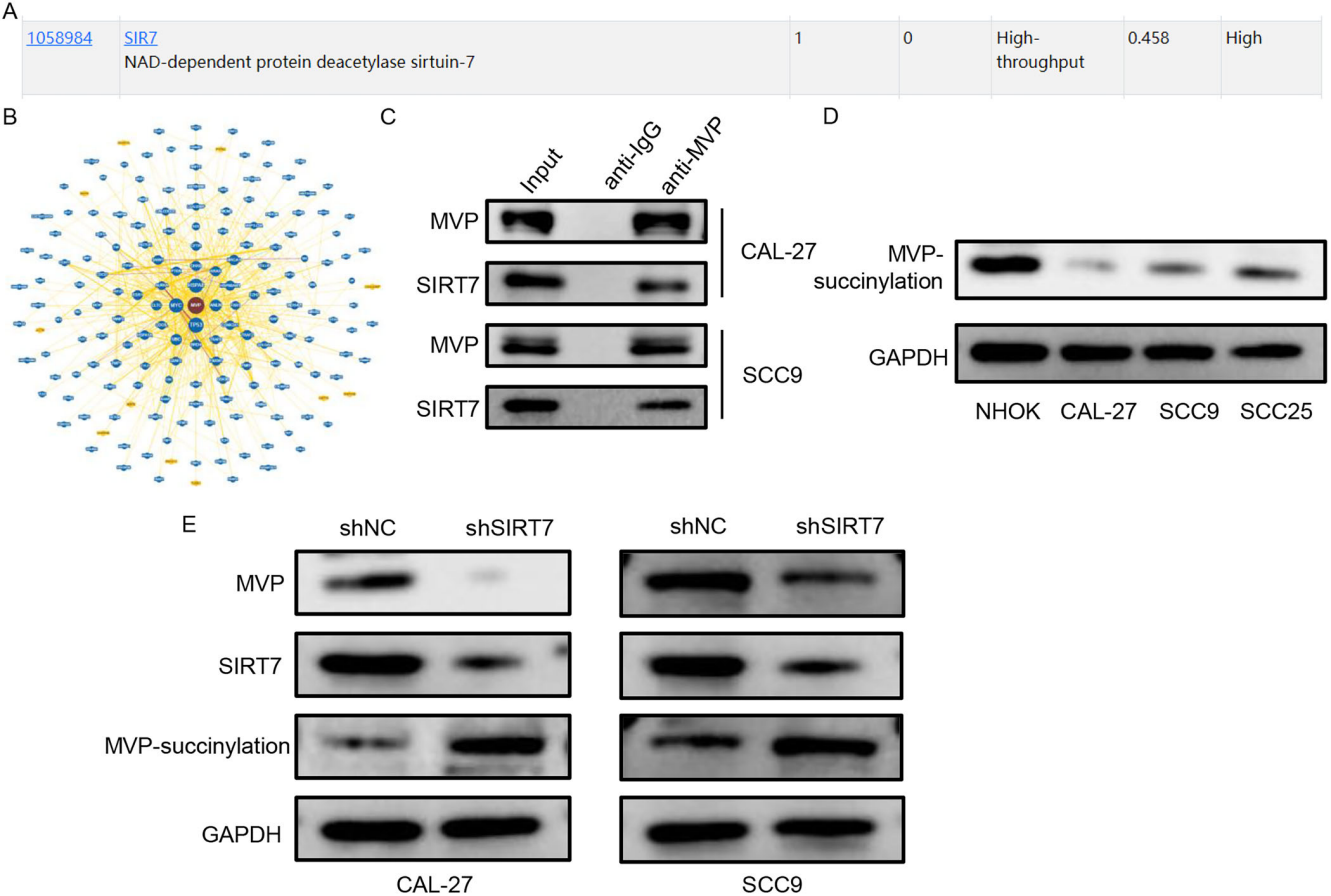
Depletion of SIRT7 enhanced MVP‐succ level in TSCC cells. (A and B) Proteins with binding potential to MVP were predicted on HitPredict and BIOGRID. (C) The interaction of MVP and SIRT7 was confirmed using co‐IP assay. (D) The succinylation level of MVP was detected in TSCC cells (CAL‐27, SCC9, and SCC25) by western blot. (E) The protein levels of MVP, SIRT7, and MVP‐ succinylation were measured by western blot in CAL‐27 and SCC9 cells. *p < 0.05; **p < 0.01; ***p < 0.001.

Proteins with binding potential to MVP were predicted on HitPredict and BIOGRID. Both HitPredict and BIOGRID showed a correlation between MVP and SIRT7 (Figure 5A,B). The interaction of MVP and SIRT7 was also verified by using Co‐IP in TSCC cells (Figure 5C). SIRT7, is a known desuccinylase located in mitochondrion. Therefore, we conjectured that MVP was succinylated in TSCC and assessed the expression of succinylation of MVP. As expounded in Figure 5D, the succinylation level of MVP in TSCC cells (CAL‐27, SCC9, and SCC25) was lower than that in NHOK cells. Besides, depletion of SIRT7 downregulated the protein expressions of MVP and SIRT7 and enhanced the expression of MVP‐succin TSCC cells (Figure 5E). These data showed that MVP was desuccinylated by SIRT7 in TSCC.

### 4.6 MVP Was Desuccinylated at K437 Site

**Figure 6:**
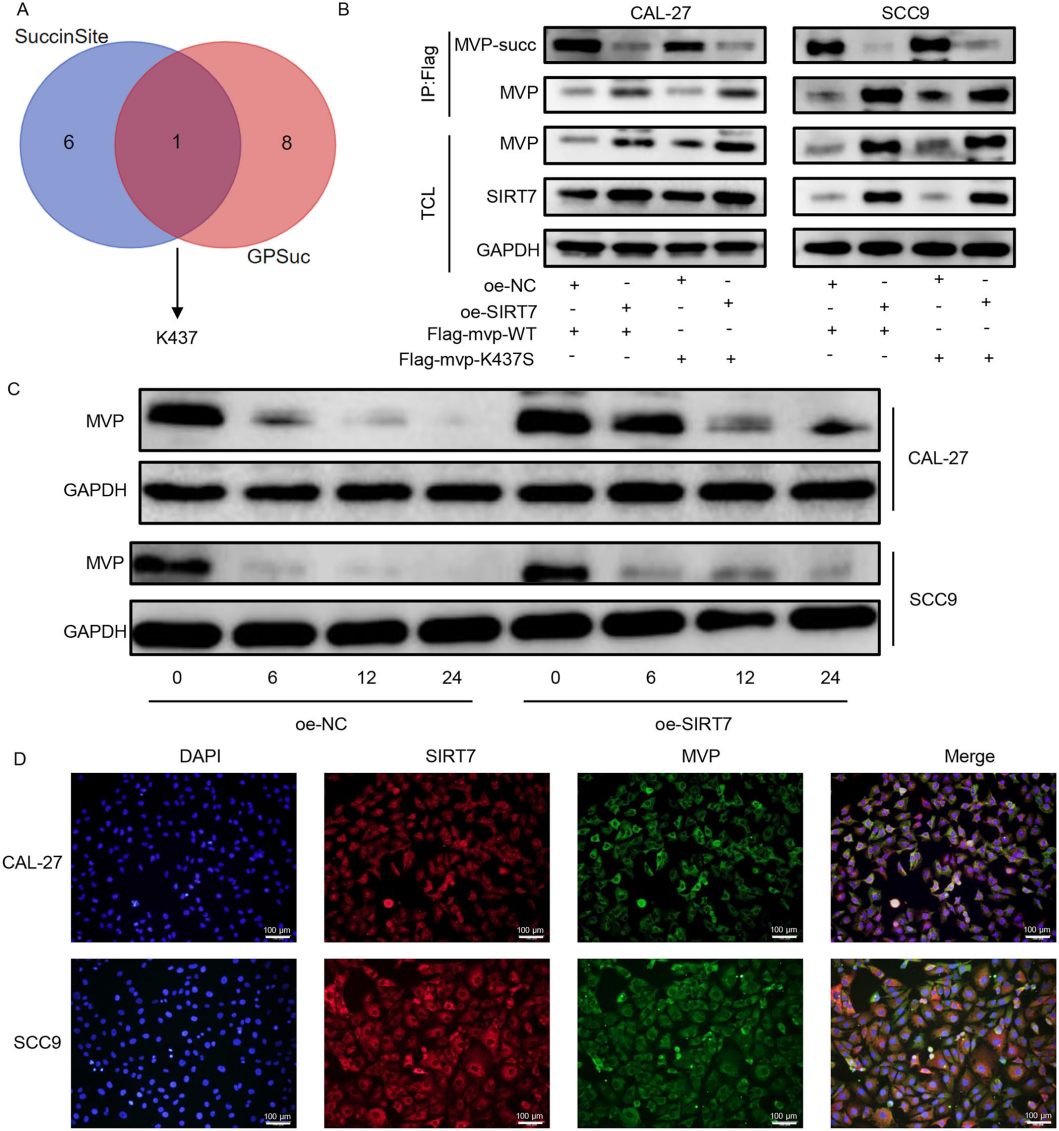
MVP was desuccinylated at K437 site. (A) The succinylation sites for MVP were predicted using the SuccinSite and GPSuc databases. (B) IP assay was performed to analyze the succinylation sites of MVP. (C) TSCC cells transfected with oe‐NC or oe‐SIRT7 vector were treated with CHX, then the protein expression of MVP was assayed by western blot at the different time points (0, 6, 12, and 24 h). (D) IF staining was utilized to assess the protein distribution of MVP and SIRT7 in TSCC cells (scale bars = 100 μm). *p < 0.05; **p < 0.01; ***p < 0.001.

SuccinSite and SuccinSite databases were utilized to predict the MVP succinylation site and the results hinted that the likely MVP succinylation site was K437 (Figure 6A). Mass spectrometry analysis showed that MVP was succinylated on K437 in CAL‐27 cells (Supporting Information S1: Figure S1). Then, IP data indicated that addition of SIRT7 declined the MVP‐succ expression and enhanced MVP level. SIRT7 suppressed the MVP‐succ level and enhanced MVP level when cotransfected with Flag‐MVP‐K437S, indicating that MVP was desuccinylated at K437 site (Figure 6B). Moreover, addition of SIRT7 upregulated the protein stability of MVP in TSCC cells (Figure 6C). IF staining was performed to confirmed the relation between MVP and SIRT7. The data demonstrated that SIRT7 colocated with MVP in TSCC cells (Figure 6D).

### 4.7 Depletion of SIRT7 Abolished TSCC Cell Malignant Behaviors Triggered by MVP

**Figure 7:**
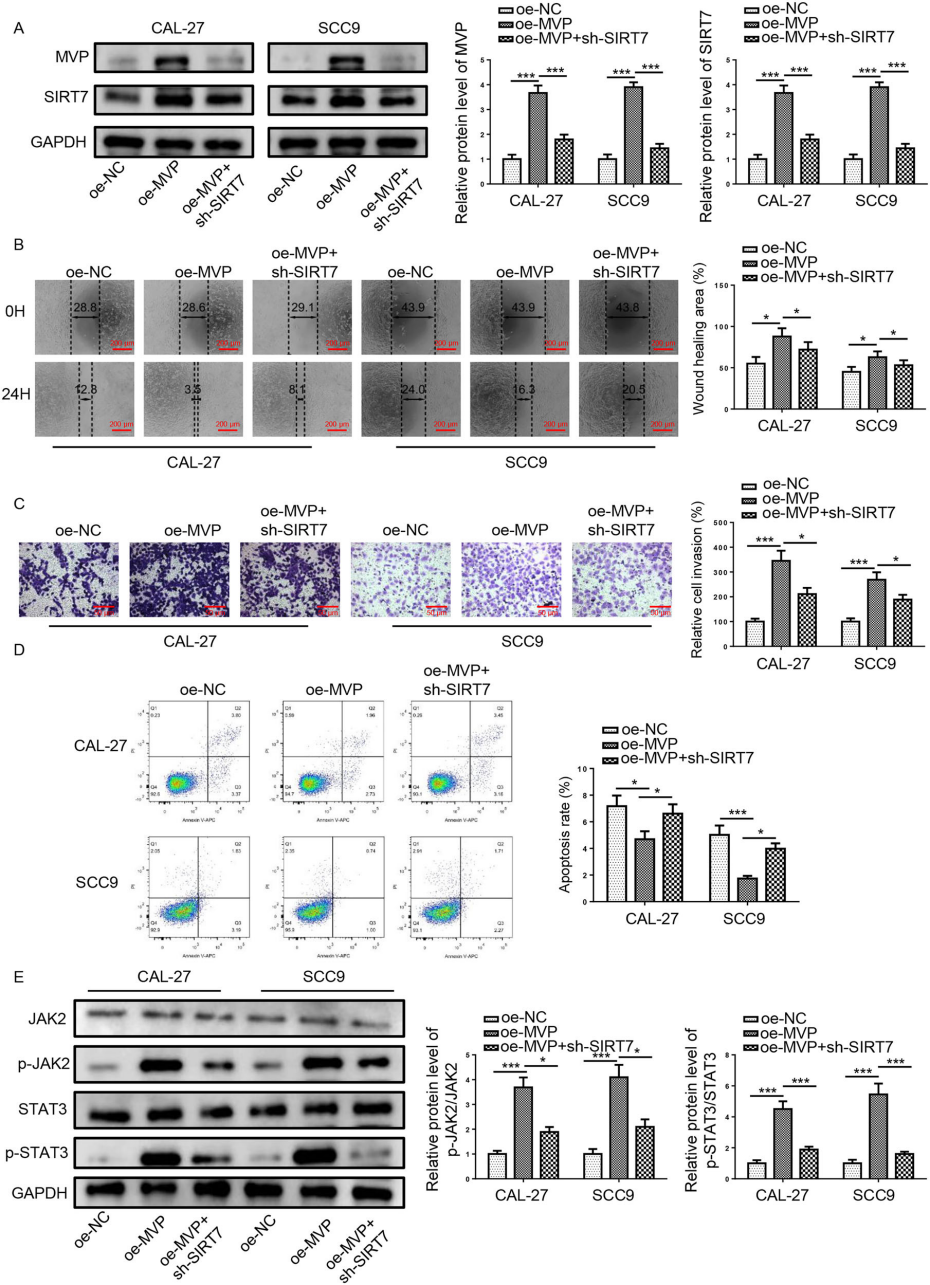
Depletion of SIRT7 abolished TSCC cell malignant behaviors triggered by MVP. The experiment was divided into the following groups: oe‐NC, oe‐MVP, oe‐MVP+sh‐SIRT7. (A) MVP and SIRT7 protein levels were measured by western blot assay. (B) migration (scale bars = 200 μm), (C) invasion (scale bars = 50 μm), and (D) apoptosis were measured by CCK‐8, wound healing, transwell, and flow cytometry assays in TSCC cells. (E) p‐JAK2, p‐STAT3, JAK2, and STAT3 protein levels were measured by western blot assay in TSCC cells. *p < 0.05; ** *p* < 0.01; *** *p* < 0.001.

To evaluate whether SIRT7 is implicated in MVP‐mediated anabatic effects on TSCC cell malignant behaviors, SIRT7 silencing in TSCC cells with or without MVP. As shown in Figure 7A, addition of MVP enhanced MVP and SIRT7 protein levels, only SIRT7 protein level was downregulated after SIRT7 silencing. Moreover, overexpression of MVP promotes cell migration (Figure 7B), and invasion (Figure 7C) and suppresses apoptosis (Figure 7D) in TSCC cells, which were abolished by SIRT7 depletion. Further, p‐JAK2 and p‐STAT3 protein levels were enhanced in MVP‐overexpressed TSCC cells, which were counteracted following SIRT7 silencing (Figure 7E). These results demonstrated that MVP promotes TSCC development via regulating SIRT7/JAK2/STAT3 axis.

## 5 Discussion

The effects of MVP on the promotion of prostate cancer (Nunes‐Xavier et al. 2023), papillary thyroid cancer (Dong et al. 2022), colorectal cancer (Pietras et al. 2021), and chondrosarcoma (Wang et al. 2021) have been substantiated in previous research. However, the role of MVP on TSCC and the underlying mechanisms involved were still unclear. Our study established a novel SIRT7‐MVP‐JAK2/STAT3 signaling axis in TSCC progression. Specifically, SIRT7 acts as a desuccinylase targeting MVP at K437, which in turn activates JAK2/STAT3 signaling to drive tumor cell proliferation, migration, and invasion. This pathway integrates posttranslational modification (succinylation), oncoprotein stabilization, and kinase signaling activation into a cohesive oncogenic network.

Major vault protein (MVP) is the principal component of vault ribonucleoprotein particles and is widely implicated in various cellular processes, including signal transduction, intracellular transport, and drug resistance (Jiang et al. 2025; Wu et al. 2023). Increasing evidence has demonstrated that MVP plays a critical oncogenic role in multiple cancer types (Wang et al. 2022a). For instance, MVP overexpression has been reported to promote proliferation and metastasis in prostate cancer (Nam et al. 2022), colorectal cancer (Pietras et al. 2021), and chondrosarcoma (Wang et al. 2021). These studies suggested that MVP acts as a facilitator of tumor progression through diverse molecular mechanisms, often linked to its ability to modulate key signaling pathways and contribute to chemoresistance. In our study, we observed significantly elevated MVP expression in both TSCC tissues and cell lines compared to normal controls, which aligns with the oncogenic profile of MVP in other cancers. Functional assays further confirmed that silencing MVP markedly suppressed TSCC cell proliferation, migration, invasion, and induced apoptosis and cell cycle arrest in vitro. Consistently, in vivo experiments demonstrated that MVP knockdown inhibited tumor growth, as evidenced by reduced tumor volume, weight, and proliferation markers. Collectively, these findings establish MVP as a crucial promoter of TSCC progression, underscoring its potential as a therapeutic target in this malignancy.

The JAK2/STAT3 signaling pathway is a critical mediator of various cellular processes, including proliferation, survival, differentiation, and immune responses (Long et al. 2024; Mengie Ayele et al. 2022). Dysregulation of this pathway has been widely implicated in the pathogenesis and progression of numerous cancers (Wang et al. 2022b; Jun et al. 2024). Aberrant activation of JAK2 leads to phosphorylation and activation of STAT3, which translocates to the nucleus and drives the transcription of genes involved in oncogenesis, such as those promoting cell cycle progression, antiapoptosis, and metastasis (Sirkisoon et al. 2018). For example, constitutive activation of JAK2/STAT3 signaling has been reported to contribute to tumor growth and metastasis in clear cell renal cell carcinoma (Deng et al. 2023), gallbladder cancer (Yang et al. 2024), and other malignancies, highlighting its role as a potential therapeutic target. In our study, we demonstrated that silencing MVP significantly suppressed phosphorylation levels of JAK2 and STAT3 in TSCC cells, indicating that MVP functions upstream of this oncogenic pathway. Importantly, activation of JAK2 by the agonist CA1 rescued the inhibitory effects of MVP knockdown on JAK2/STAT3 phosphorylation and reversed the suppression of malignant behaviors such as proliferation, migration, invasion, and apoptosis in TSCC cells. These findings provide strong evidence that MVP promotes TSCC progression, at least in part, through activation of the JAK2/STAT3 signaling axis. This newly uncovered MVP/JAK2/STAT3 regulatory mechanism adds to the understanding of TSCC tumorigenesis and suggests that targeting this pathway may hold therapeutic potential.

SIRT7 belongs to the sirtuin family of NAD⁺‐dependent deacetylases and desuccinylases, which are well known to regulate diverse cellular processes, including metabolism, DNA repair, gene expression, and stress responses (Li et al. 2024). Among the seven mammalian sirtuins, SIRT7 is predominantly localized in the nucleus and mitochondria, and has emerged as a crucial modulator of posttranslational modifications such as lysine acetylation and succinylation (Park et al. 2017). Lysine succinylation is a relatively recently identified acylation modification that significantly alters protein charge and structure, thereby influencing protein stability, enzymatic activity, and interactions (Park et al. 2017). Sirtuins, particularly SIRT5 and SIRT7, act as key desuccinylases, removing succinyl groups from target proteins to modulate their function (Xia et al. 2022, 2022; Ge et al. 2022). Recent studies have highlighted the role of SIRT7‐mediated desuccinylation in cancer biology. For example, SIRT7 has been shown to repress succinylation of KIF23, affecting cell cycle progression and tumor growth (Wu et al. 2024). By regulating succinylation levels on specific substrates, SIRT7 influences oncogenic signaling pathways and contributes to tumor cell survival and proliferation. However, the detailed mechanisms by which SIRT7 modulates succinylation in TSCC remain poorly understood. In our study, we identified MVP as a novel substrate of SIRT7‐mediated desuccinylation in TSCC cells. Bioinformatic predictions coupled with co‐IP assays confirmed the interaction between SIRT7 and MVP. We observed that MVP succinylation levels were lower in TSCC cells compared to normal oral keratinocytes, consistent with increased desuccinylation activity. Knockdown of SIRT7 resulted in elevated MVP succinylation and reduced MVP protein levels, suggesting that SIRT7 stabilizes MVP through desuccinylation. Mass spectrometry further pinpointed lysine 437 (K437) as the specific desuccinylation site on MVP, and mutation analysis demonstrated that SIRT7 directly targets this residue to regulate MVP stability.

In summary, SIRT7‐mediated MVP desuccinylation facilitates proliferation, migration, and invasion and inhibits apoptosis of TSCC cells by activating JAK2/STAT3 pathway. Our study provides a new idea for the clinical treatment of TSCC.

## Ethics Statement

The study was approved by the Ethics Committee of Stomatological Hospital of Shenyang.

## Supporting information

Figure S1: Mass spectrometric verification of MVP succinylation at K437.

## Data Availability

The datasets used and/or analyzed during the current study are available from the corresponding author on reasonable request.
