# Supplementary figures and images for: SIRT7‐Mediated MVP Desuccinylation Facilitates Tongue Squamous Cell Carcinoma Progression by Activating JAK2/STAT3 Pathway
Source: Cell Biol Int. 2025 Jun 30;49(9):1184–96. doi: 10.1002/cbin.70048 (PMC12340476; doi:10.1002/cbin.70048)

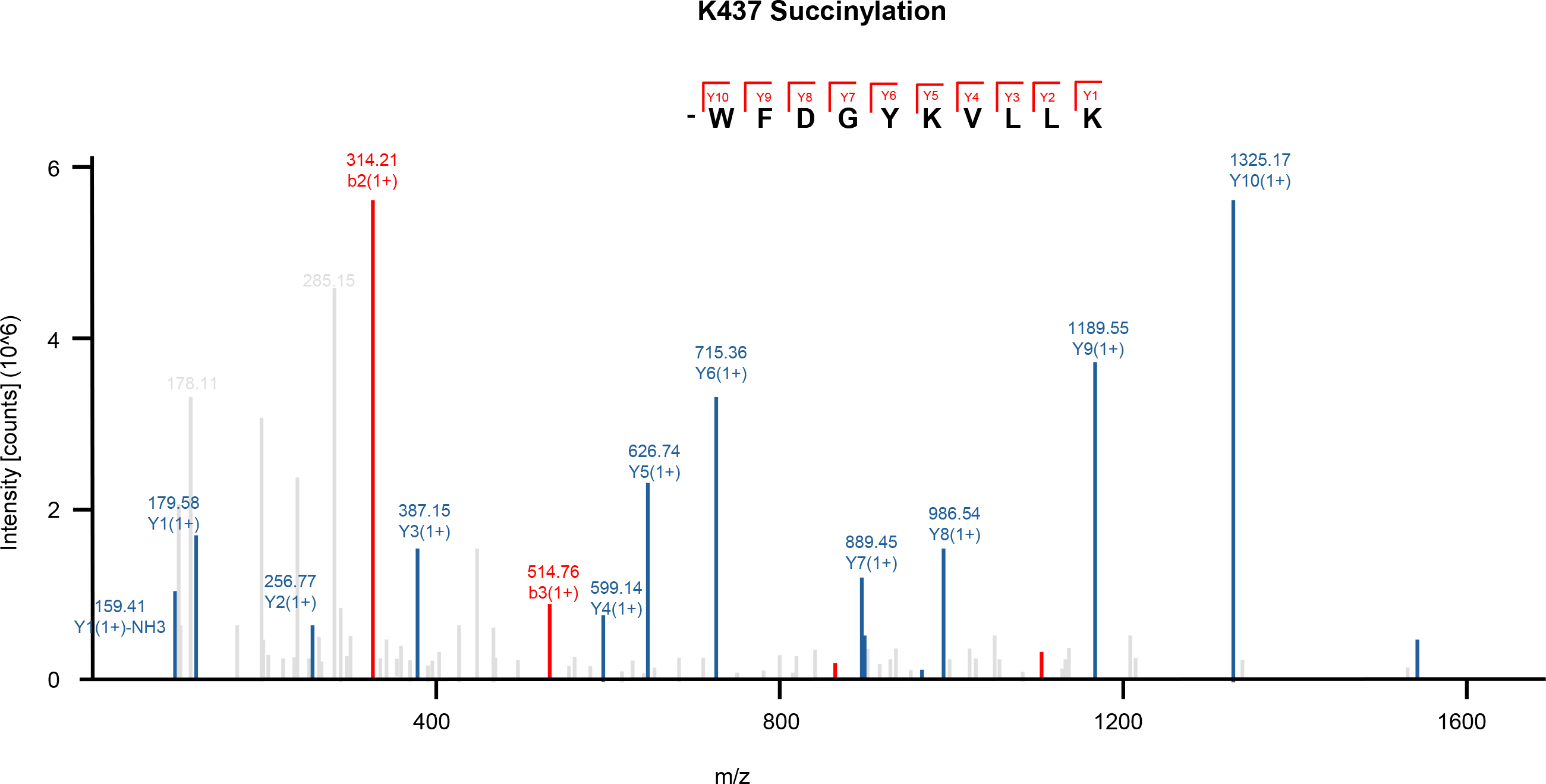

Supplement: Supplementary file 1 — Figure S1: Mass spectrometric verification of MVP succinylation at K437. [file CBIN-49-1184-s001.tif]
